# CAG Repeat Instability in the Peripheral and Central Nervous System of Transgenic Huntington’s Disease Monkeys

**DOI:** 10.3390/biomedicines10081863

**Published:** 2022-08-02

**Authors:** In K. Cho, Faye Clever, Gordon Hong, Anthony W. S. Chan

**Affiliations:** 1 Division of Neuropharmacology and Neurologic Diseases, Yerkes National Primate Research Center, Emory University, Atlanta, GA 30322, USA; awschan@hotmail.com; 2 Department of Human Genetics, Emory University School of Medicine, Atlanta, GA 30322, USA; fhclever1@gmail.com (F.C.); ghong@neomed.edu (G.H.)

**Keywords:** Huntington’s disease, trinucleotide repeats, central nervous system, peripheral system, transgenic monkey model

## Abstract

Huntington’s Disease (HD) is an autosomal dominant disease that results in severe neurodegeneration with no cure. HD is caused by the expanded CAG trinucleotide repeat (TNR) on the Huntingtin gene (*HTT*). Although the somatic and germline expansion of the CAG repeats has been well-documented, the underlying mechanisms had not been fully delineated. Increased CAG repeat length is associated with a more severe phenotype, greater TNR instability, and earlier age of onset. The direct relationship between CAG repeat length and molecular pathogenesis makes TNR instability a useful measure of symptom severity and tissue susceptibility. Thus, we examined the tissue-specific TNR instability of transgenic nonhuman primate models of Huntington’s disease. Our data show a similar profile of CAG repeat expansion in both rHD1 and rHD7, where high instability was observed in testis, liver, caudate, and putamen. CAG repeat expansion was observed in all tissue samples, and tissue- and CAG repeat size-dependent expansion was observed. Correlation analysis of CAG repeat expansion and the gene expression profile of four genes in different tissues, clusterin (*CLU*), transferrin (*TF*), ribosomal protein lateral stalk subunit P1 (*RPLP1*), and ribosomal protein L13a (*RPL13A*), showed a strong correlation with CAG repeat instability. Overall, our data, along with previously published studies, can be used for studying the biology of CAG repeat instability and identifying new therapeutic targets.

## 1. Introduction

Huntington’s Disease (HD) is an autosomal dominant disease that results in severe neurodegeneration and cognitive, behavioral, and motor decline, followed by death approximately 10–15 years after diagnosis [1,2,3]. HD is caused by the expanded CAG trinucleotide repeat (TNR) on the Huntingtin gene (*HTT*) [1,4], which results in a longer polyglutamine (polyQ) chain and misfolding of the huntingtin (HTT) protein. The expansion of CAG repeat tract in exon 1 of *HTT* results in expanded polyglutamine-containing fragments that form aggregates in the cell [5]. Huntingtin is a large protein (>340 kDa) with large a-helical HEAT (huntingtin, elongation factor 3, protein phosphatase 2A, and lipid kinase TOR) repeat protein [6]. The expansion results in oligomerization disrupting cellular functions and impairing proteostasis, eventually resulting in alterations or neural functions [5,7,8]. 

The functions of wild-type (WT) HTT, though not fully understood, are essential in neurogenesis and the prevention of cell death [9,10]. The underlying mechanisms of TNR expansion remain largely unknown [11], with much interest residing in DNA repair pathways [12,13,14,15,16]. The pathogenic properties of mutant huntingtin (mHTT) itself likely arise as a result of mHTT fragment aggregation in the nuclei of cells [17], with exon 1 fragments being the most toxic due to their small size [17,18,19,20,21].

The tissue most affected by HD is the striatum, composed of the caudate nucleus and putamen, with high TNR instability and protein aggregation [22,23,24]. Sperm and most brain areas also show high TNR instability, while some studies showed testes and cerebellum are relatively unaffected [22,23,25]. The liver has also been shown to have high instability of TNR in HD mice, which mirrors peripheral symptoms such as weight loss seen in human HD patients [13,23,25,26,27]. Most peripheral tissues such as the adrenal gland, spleen, pancreas, lung, heart, and kidneys generally showed more stable TNR [23,25,26,28,29].

Increased CAG repeat length is associated with a more severe phenotype, greater TNR instability, and earlier age of onset [25,30,31,32,33,34]. The full mutation length is over 40 repeats, but more than 70 repeats result in juvenile HD [1,3], typically inherited paternally due to the large TNR expansions that occurred during spermatogenesis [34,35]. Much evidence points to mHTT as the driver of HD pathology rather than the result or byproduct. In the absence of symptoms, non-pathogenic CAG repeats in mice still expand, implying that the instability of CAG repeats is not a result of disease [36]. There was a considerable delay in the onset of symptoms in HD mice lacking somatic instability compared to HD mice with typical mosaicism [37,38]. Furthermore, the presence of N-terminal mHTT fragments in the nuclei of cells leads to apoptosis [39], and disease progression can be accelerated solely by increasing the mHTT concentration in the nuclei of cells [18]. Although expanded polyglutamine plays an important role in the pathogenesis of HD, a recent study has shown that uninterrupted *HTT* CAG repeat size determines the onset of HD [40]. Additionally, the same study identified polymorphic variation at multiple DNA maintenance genes that are associated with somatic repeat expansion [40]. Thus, the direct relationship between CAG repeat length and molecular pathogenesis makes TNR instability a useful measure of symptom severity and tissue-specificity.

Unfortunately, one major obstacle in the study of HD is the lack of animal models that reflect the robust symptoms displayed in human HD patients. Because the aging process differs between small and large animals (e.g., mice and monkeys), mHTT affects mice differently, causing them to display phenotypes different from HD patients, such as weight gain in YAC128 and BACHD mice [41] as opposed to weight loss [42]. Additionally, instability in peripheral tissues such as the liver is less consistent among mouse models [27,43]. While mouse models have been critical for advancing HD research, they are nonetheless limited biologically in their capacity to mirror complex human pathology. To further advance our understanding of HD pathogenesis, it is necessary to use large animal models that better replicate HD pathology and progression for studying HD pathogenesis, developing potential treatment, and preclinical studies. The genetic and physical similarities between humans and nonhuman primates have allowed us to model the human-specific pathology [42,44,45,46,47]. Thus, we examine the tissue-specific TNR instability of two HD monkeys [48] and compare them with humans. The HD monkey model developed pathology mirroring human HD, such as decreases in the levels of neuronal health marker N-acetyl aspartate (NAA) and white matter abnormalities in the corpus callosum, caudate, and primary motor cortex [27,45,49]. They also displayed progressive HD symptoms not observed in some mouse models, such as seizures, striatal atrophy, chorea, and dystonia [49]. In our monkey model (rHD1 and rHD7), both monkeys showed mHTT aggregated in both the caudate nucleus and putamen at age of five [49]. rHD1, with a longer CAG repeat tract length, showed more intranuclear inclusions compared with rHD7, which showed more sparsely stained cells throughout the brain [49]. 

Somatic mosaicism is well documented in animal models [13,25,26,50,51,52] and human postmortem studies [27,30,53]. We recently reported longitudinal progressive CAG repeat expansion in peripheral blood cells and sperm of HD monkeys [54]. We observed a similar pattern in CAG repeat size and age-dependent CAG repeat expansion [54] between HD monkey and rodent models [25,26]. However, CAG repeat expansion was observed in HD monkey sperm, while a limited expansion was observed in some HD rodent models. 

The aim of this study was to further investigate tissue or cell-type specificity of CAG expansion in our transgenic HD monkeys and investigate whether our model recapitulates human pathology. We examined CAG repeat instability of postmortem tissues of transgenic HD monkeys and investigated proteins that are expressed in various proteins that show a correlation with CAG repeat expansion which could lead to new insight into the underlying mechanisms of CAG repeat instability and expansion. 

## 2. Materials and Methods

Animals: Four *Rhesus macaques*, rHD1, rHD7, and two WTs, were used in this experiment. The two HD monkeys (rHD 1 and rHD7) carried transgenes with different length of CAG repeats in exon 1 of *HTT* regulated by human ubiquitin C promoter and human *HTT* gene promoter, respectively. Both monkeys were euthanized at five years of age [45,46,55].

Brain tissues (caudate nucleus, cerebellum, hippocampus, motor cortex thalamus, prefrontal cortex, and putamen) and peripheral tissues (adrenal gland, heart, kidney, liver, lung, pancreas, and testes) were collected, snap-frozen in liquid nitrogen, and stored at −80 °C until analysis. 

DNA Isolation: An approximately 0.5 cm^3^ sample of tissue was used for DNA extraction. DNA extraction was completed using a Maxwell^®^ 16 Tissue DNA Purification Kit (Promega, Madison, WI, USA). The concentration and purity of DNA extractions were measured using NanoDrop™ 2000 (ThermoFisher, Waltham, MA, USA).

PCR: For the PCR, we used 500 ng of genomic DNA, 0.4 µM forward primer (HD32G; 5′-FAM-CTACGAGTCCCTCAAGTCCTTCCAGC), 0.4 µM reverse primer (MD177R; 5′-GACGCAGCAGCGGCTGTGCCTG), 1× Takara PCR buffer, 1 mM deoxynucleotide triphosphate (dNTP), 0.5 U Takara Taq polymerase, and 4 µM Betaine. The PCR protocol was set using the following: cycling conditions were 98 °C for 5 min, 40 cycles of 95 °C 5 min, 67 °C 0.75 min, 72 °C 1.5 min, followed by 10 min at 72 °C.

Data Analysis: PCR product was sent to Emory Integrated Genomics Core for GeneScan analysis. In total, 1.5 µL of PCR products were mixed with 0.5 µL of GeneScan™ 500 ROX™ (ThermoFisher, Waltham, MA, USA) and 9.5 µL of Hi-Di™ Formamide (Applied Biosystems). Samples were denatured at 95 °C for 5 min and ran on a 3130xl Genetic Analyser (Applied Biosystems). The data was analyzed using GeneMarker^®^ (SoftGenetics). From the electrograms, only peaks with a height above 10% of the highest peak were included in calculations. Expansion index was calculated by modifying instability index [13] following the equation:

$$\Sigma \left(\left(\frac{\mathrm{peak}\;\mathrm{height}}{\Sigma \mathrm{peak}\;\mathrm{heights}}\right)\left(\Delta \mathrm{TNR}\;\mathrm{from}\;\mathrm{the}\;\mathrm{reference}\;\mathrm{allele}\right)\right)$$

For the expansion index, instead of calculating changes from the modal peak (i.e., the highest peak), reference tissue with the most stable CAG repeat (muscle in both rHD1 and rHD7) was used to calculate the changes, which was multiplied by the normalized peak height (peak height/∑peak height). The sum of all values was expressed as an expansion index. The expansion index represents the instability of a sample and its tendency towards expansions (i.e., positive values) or contractions (i.e., negative values). The expansion index close to zero indicates low instability. Positive values indicate expansions, and negative values indicate contractions. 

For curve-fit data analysis, masked allele data was imported into MATLAB (The MathWorks Inc., Natick, MA, USA). The curve-fitting was processed as previously described [28]. Briefly, imported data was analyzed with the ipf.m function in MATLAB, minimizing error to under 5% and maximizing the overall fitness R^2^ value to greater than 0.95. The Gaussian distribution was used to fit the curves due to the nature of the data. Later, curve-fit data and the electropherogram were superimposed using Adobe Illustrator (Adobe). All the data with mean, error, and R^2^ values are presented in the Supplemental Tables (Table S1 and Table S2). 

Statistical analysis: All curve-fit data with the position of mean, error, and R^2^ value are presented in the Supplemental Tables. Individual alleles (red curves) were plotted in the box plot to deconvolute individual alleles that arise from the parental alleles since different tissues have different expansion profiles, i.e., continuous in the tail and periodic expansion in striatum [28]. Continuous expansion conforms to a random bi-directional forward-biased model while dramatic expansion demonstrates periodicity of inserting stable TNR segments occurring within small cell populations causing the subsequent cell population to have similar repeat length within the normal distribution [28]. Therefore, the curve-fit method was used to deconvolute the alleles that arise from periodic expansion within the electropherograms. For the linear regression, the Pearson correlation coefficient (R^2^) and statistical significance (*p*-value) were calculated using GraphPad Prism Version 8.0.2 (GraphPad Software, La Jolla, CA, USA). For all correlation analyses, one-tailed Spearman’s correlation analysis was conducted on GraphPad Prism.

Correlation Data Analysis: To characterize the TNR-associated gene expression, we extracted gene expression data from the tissue used in this study from Genotype-Tissue Expression (GTEx) project [55]. From the top 100 expressed genes among tissues with high TNR instability, liver, testis, caudate, and putamen, a total of 35 genes were commonly expressed in all four tissues. The median transcripts per million (TPM) data were downloaded from the database, and all 35 genes were tested for correlation with the expansion index and curve-fit range data. The correlation was tested using corrplot in R. Of 35 genes, only 4 showed either significantly positive or negative correlation with either expansion index or curve-fit range data (*CLU*, *TF*, *RPL13A*, and *RPLP1*). Gene expressions from *NEIL1* and *MSH3* were added as a reference. The median TPM and either expansion index or curve-fit range were plotted, and correlations between the two were calculated. For all correlation analyses, one-tailed Spearman’s correlation analysis was conducted on GraphPad Prism (GraphPad 8.0.2).

## 3. Results

Tissue samples were collected from two WT monkeys and two HD monkeys (rHD1 and rHD7; Table 1). 

**Figure 1:**
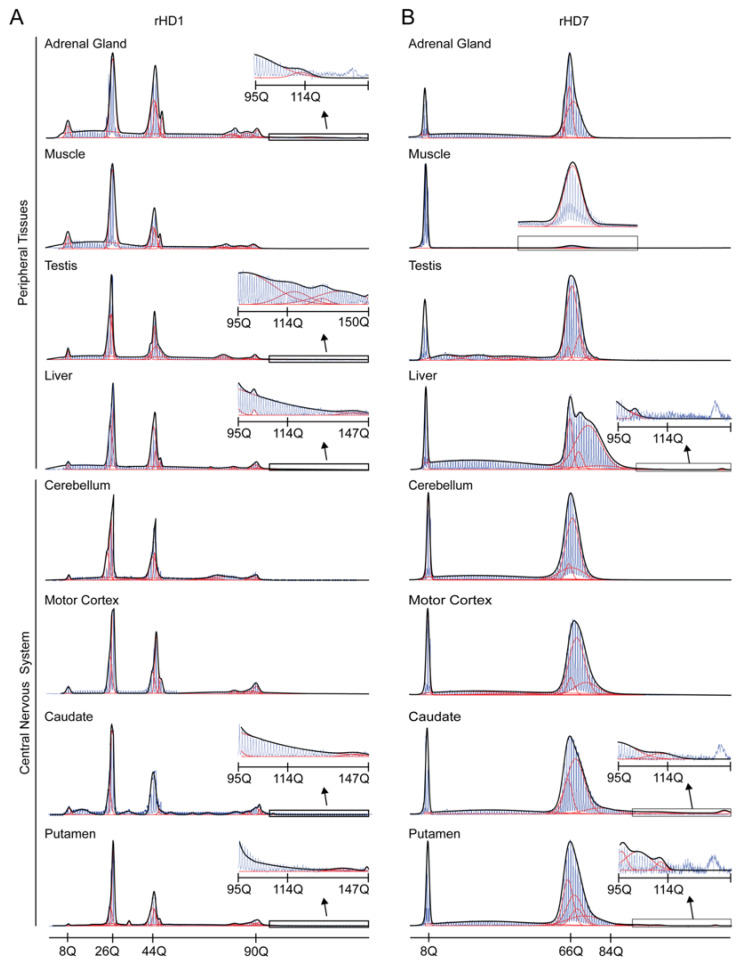
Representative electropherograms superimposed with curve-ft results of rHD1 and rHD7. (**A**) In rHD1, testis and liver showed the highest expansion among peripheral tissue samples, while caudate and putamen showed the highest expansion among the central nervous system tissue samples. (**B**) In rHD7, the liver showed the highest expansion among the peripheral tissue samples, and caudate and putamen showed the largest expansion among the central nervous system tissue samples. In both rHD1 and rHD7 tissue samples, the muscle was the most stable tissue samples among all tissue samples collected and used as a reference when calculating the expansion index. Red lines represent individual curves and black lines represent overall curve-fit results.

Both HD monkeys were created by injecting lentivirus vectors into oocytes. rHD1 was created with a vector expressing exon 1 of the human *HTT* gene with 84 CAG repeats, and rHD7 was created with a vector expressing exons 1–10 of the human *HTT* with approximately 67–72 CAG repeats under the human *HTT* promoter [48,56]. Although the integration sites and precise copy numbers of transgenes were not analyzed, lymphocytes from 3 month-old rHD1 showed mutant alleles at 27Q, 44Q, 76Q, and 87Q [57], and lymphocytes from 12 month-old rHD7 showed mutant allele at 66Q [47]. Both monkeys were euthanized at the age of five. DNA was extracted from the tissues and then underwent PCR specifically targeting the CAG repeats of normal and mutant *HTT* genes. Representative electropherograms and curve-fit data of several notable tissues from rHD1 and rHD7 are shown in Figure 1. 

**Figure 2:**
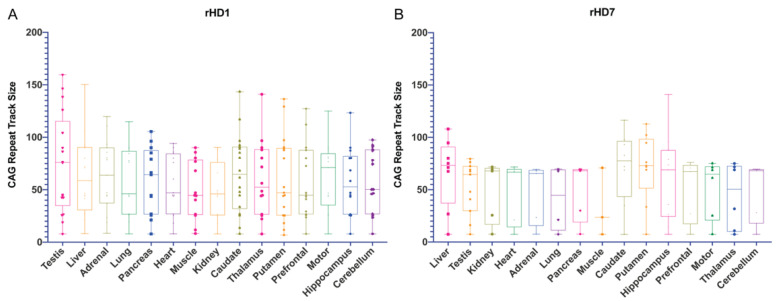
Curve-fit data of rHD1 and rHD7 arranged from the tissues with the highest CAG expansion to most stable. (**A**) Testis showed the largest CAG expansion and showed the emergence of multiple large alleles derived from 77Q. The kidney showed little emergence of larger alleles among peripheral tissue samples. Among the central nervous tissue samples, caudate, thalamus, and putamen showed similar emergency of larger alleles derived from 77Q. (**B**) Liver showed the largest CAG expansion followed by the testis in rHD7 among the peripheral tissue samples. Other peripheral tissue samples showed relatively stable CAG size while the muscle was the most stable. The caudate, hippocampus, and putamen showed a large CAG expansion among the central nervous samples, while the other samples were stable.

All electropherograms are provided in supplemental data (Figures S1–S4). The curve-fit method was used in this study to capture multiple alleles derived from the primary allele following the method described by Mollersen et al. [28], which was used in our previous studies [54,57]. The result of the curve-fit data is provided in supplemental data with the error and goodness of fit value (R^2^) (Supplemental Tables S1 and S2). The electropherograms show the mosaicism of CAG repeats with different repeat lengths. From these electrograms, peak sizes from the curve-fit data and expansion index were used in further analysis. Curve-fit data show that the liver showed a larger range of allele sizes in peripheral tissues denoting the high instability in these tissues in both rHD1 and rHD7 (Figure 2A,B).

In rHD1, adrenal gland, lung, and pancreas showed moderate instability while heart, muscle, and kidney were relatively stable (Figure 1A and Figure 2A). In the central nervous system (CNS), tissue samples showed relatively high instability across all brain regions except the cerebellum, while caudate, thalamus, and putamen showed the highest instability (Figure 1A and Figure 2A). In rHD 7, liver, caudate, hippocampus, and putamen showed a broad range of alleles, while the rest of the tissues were relatively stable in allele sizes (Figure 1B and Figure 2B). A similar trend was observed in both rHD1 and rHD7 where high instability was observed in liver and caudate and putamen in the larger allele (Figure 1 and Figure 2). Among the tissues from the CNS, a large median value with high CAG mosaicism was observed in caudate and putamen of rHD1 and rHD7 (Figure 2 and Figure S5). 

**Figure 3:**
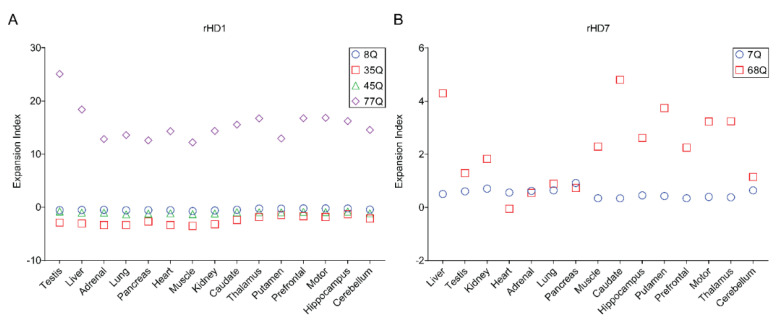
Expansion indexes of rHD1 and rHD7 peripheral and central nervous system samples are arranged in the same order as the curve-fit data. (**A**) All alleles, except 77Q, were stable. Like curve-fit data, testis and liver showed the highest instability compared to the rest of the peripheral tissue samples, although 77Q in all peripheral tissue samples showed a high expansion index. Among the central nervous system samples, 77Q alleles in all tissue samples showed similar instability. (**B**) All peripheral tissue samples, except the liver, showed similar instability in both 7Q and 68Q. In the central nervous system, the caudate showed the highest instability, followed by the putamen.

The expansion index was also calculated by modifying the instability index [13]. All WT tissues had 0 expansion indexes suggesting the lack of any CAG repeat expansions in small repeat sizes. The expansion indexes were plotted according to reference alleles (8, 35, 45, and 77Q for rHD1; 7 and 68Q for rHD7) (Figure 3).

Similar to curve-fit data, testis and liver showed high instability in the 77Q allele in rHD1 (Figure 3A). In all tissues, 77Q showed relatively high instability (Figure 3A). In rHD7, liver and all central nervous system tissue samples, except cerebellum, showed high instability (Figure 3B). 

**Figure 4:**
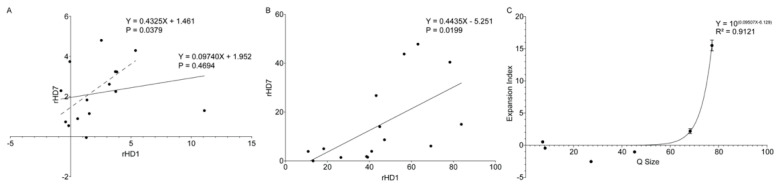
Correlations of expansion indexes between samples. (**A**) Expansion indexes from each tissue from rHD1 and rHD7 were plotted to show the tissue specificity of CAG expansion. A Spearman’s correlation showed a strong positive correlation between rHD1 and rHD7, which was statistically significant (R_s_(14) = 0.5341, *p* = 0.0261) when the rHD1 testis index was removed due to high expansion of CAG repeats in rHD1 testis. (**B**) The range of curve-fit data was plotted for corresponding tissue samples. A strong positive correlation was found between rHD1 and rHD7 (R_s_(14) = 0.5926, *p* = 0.0199). (**C**) When all expansion indexes of rHD1 and rHD7 are plotted against CAG repeat size (Q size), the expansion index follows a nonlinear regression model where the expansion index increases exponentially around 60Q.

Spearman’s correlation test was used to determine whether CAG expansion depends on tissue type (i.e., tissue specificity) or the size of CAG repeat (i.e., size specificity) (Figure 4). 

When expansion indexes were plotted for rHD1 and rHD7 for each correlating tissue, a strong positive correlation was observed between rHD1 and rHD7 with statistical significance (R_s_(14) = 0.5341, *p* = 0.0379) when rHD1 testis was excluded after Grubbs’ outlier test (α = 0.05, G = 2.858) (Figure 4A). The range of the curve-fit data also showed a strong positive correlation between rHD1 and rHD7 (R_s_(14) = 0.5926, *p* = 0.0190) (Figure 4B). When all expansion indexes of rHD1 and rHD7 were plotted against CAG repeat size (Q size), the expansion index followed a nonlinear regression model (R^2^ = 0.9121). The expansion index increases exponentially around 60Q (Figure 4C). 

**Figure 5:**
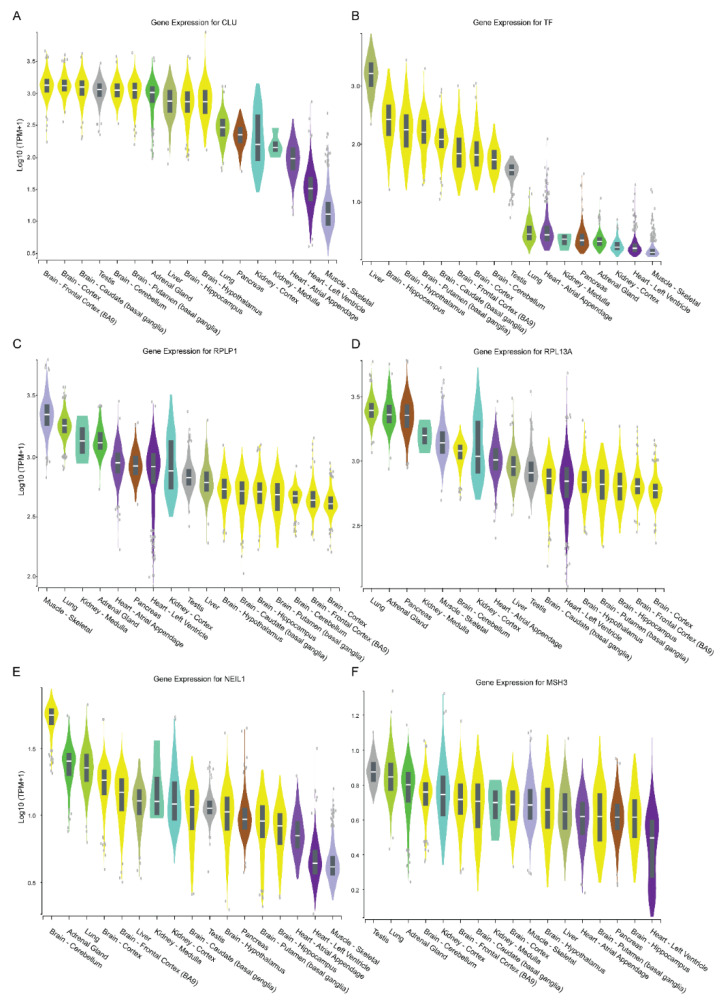
The genotype-tissue expression (GTEx) profile of the selected genes from the organs used in this study. Among tissues with high CAG repeat instability (liver, testis, caudate, and putamen), a list of 100 genes highly expressed in each organ was analyzed on the GTEx database. Among 35 genes in all four tissue samples, genes showing a similar trend as the CAG instability were selected for further analysis (*CLU*, *TF*, *RPLP1*, and *RPL13A*). (**A**,**B**) *CLU* and *TF* expression showed a similar trend as the expansion index where liver, testis, and brain showed a high gene expression while muscle and heart showed a low gene expression. (**C**,**D**) *RPLP1* and *RPL13A* showed the opposite trend as the expansion index. (**E**,**F**) Among the genes associated with CAG expansion, *NEIL1* and *MSH3* gene expressions were plotted.

To further determine the factors contributing to tissue specificity of CAG expansion, lists of the top 100 expressed genes in four tissues with high instability (liver, testis, caudate, and putamen) were retrieved from the Genotype-Tissue Expression (GTEx) database (Figure S6). Among the 35 genes that were common in all four tissues, genes with expression patterns similar to the trend of CAG instability were selected for further analysis (clusterin (*CLU*), transferrin (*TF*), ribosomal protein lateral stalk subunit P1 (*RPLP1*), and ribosomal protein lateral stalk subunit P1 (*RPL13A*)) (Figure 5).

**Figure 6:**
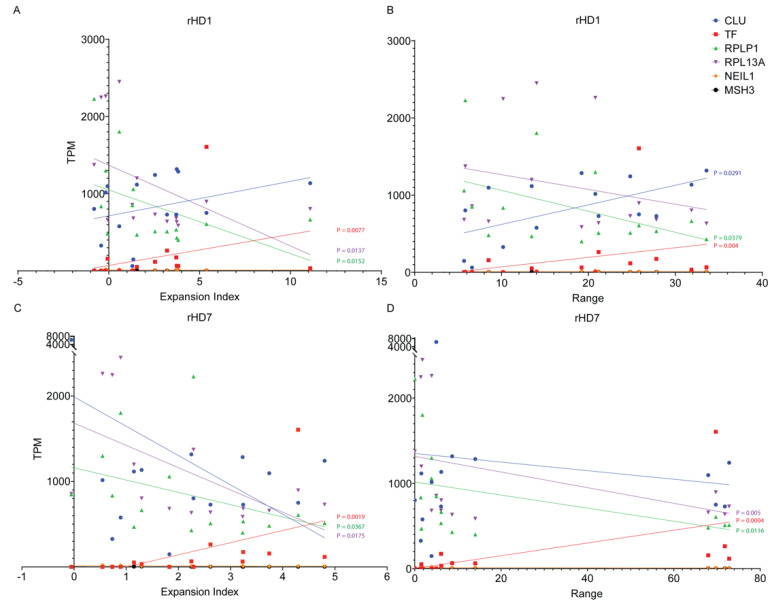
Correlation of gene expression in tissue samples used in this study against expansion index and curve-fit data. (**A**) A Spearman’s correlation analysis of rHD1 data showed a strong positive correlation between expansion index and TF expression in rHD1, which was statistically significant (R_s_(14) = 0.6214, *p* = 0.0077) while CLU showed positive correlation that was close to statistical significance (R_s_(14) = 0.4321, *p* = 0.0547). RPLP1 and RPL13A expression levels showed a statistically significant strong negative correlation with the expansion index ((R_s_(14) = −0.5750, *p* = 0.0137) and (R_s_(14) = −0.5648, *p* = 0.0152), respectively). NEIL1 and MSH3 did not show any significant correlation. (**B**) The Spearman’s correlation showed a strong positive correlation between range of the curve fit data with CLU and TF expression, which was statistically significant ((R_s_(14) = 0.5036, *p* = 0.0291) and (R_s_(14) = 0.6679, *p* = 0.004), respectively). RPLP1 expression level showed a statistically significant strong negative correlation with the expansion index (R_s_(14) = −0.4750, *p* = 0.0379). RPL13A, NEIL1, and MSH3 did not show any significant correlation. (**C**) The Spearman’s correlation of rHD7 expansion index data showed a statistically significant positive correlation between expansion index and TF expression (R_s_(14) = 0.7143, *p* = 0.0019) while no significant correlation was observed with CLU expression (R_s_(14) = 0.3214, *p* = 0.4566). Both RPLP1 and RPL13A showed statistically significant negative correlations ((R_s_(14) = −0.4786, *p* = 0.0367) and (R_s_(14) = −0.5523, *p* = 0.0175), respectively). No significant correlation was found for both NEIL1 and MSH3. (**D**) When a Spearman’s correlation was analyzed with range of curve-fit data and gene expression, similar correlation trends were observed as expansion index. A statistically significant positive correlation between curve-fit range and TF expression (R_s_(14) = 0.7893, *p* = 0.0004) was observed while no significant correlation was observed with CLU expression (R_s_(14) = 0.3607, *p* = 0.0935). Both RPLP1 and RPL13A showed a statistically significant negative correlation ((R_s_(14) = −0.5893, *p* = 0.0116) and (R_s_(14) = −0.6524, *p* = 0.005), respectively). No significant correlation was found for both NEIL1 and MSH3.

*CLU* and *TF* were highly expressed in the testis, liver, and brain where CAG instability was high (Figure 5A,B). Two genes (*RPLP1* and *RPL13A*) showed the opposite trend as the CAG expansion index (Figure 5C,D). Among the genes previously that have been associated with CAG expansion in HD, *NEIL1* [58] and *MSH3* [59,60] were also investigated to see whether their expression correlates with CAG instability (Figure 5D,E). To further investigate the correlation between gene expression patterns and CAG instability in different tissues, we plotted gene expression data (transcripts per million (TRM)) against the expansion index and curve-fit data of corresponding tissue (Figure 6). 

rHD1 data showed a strong positive correlation between expansion index and *TF* expression in rHD1, which was statistically significant (R_s_(14) = 0.6214, *p* = 0.0077) while *CLU* showed a positive correlation that was close to statistical significance (R_s_(14) = 0.4321, *p* = 0.0547) (Figure 6A). *RPLP1* and *RPL13A* expression level showed a statistically significant strong negative correlation with the expansion index ((R_s_(14) = −0.5750, *p* = 0.0137) and (R_s_(14) = −0.5648, *p* = 0.0152), respectively) (Figure 6A). However, *NEIL1* and *MSH3* did not show any significant correlation (Figure 6A). On the other hand, a strong positive correlation ((R_s_(14) = 0.5036, *p* = 0.0291) and (R_s_(14) = 0.6679, *p* = 0.004)) between the range of the curve fit data with *CLU* and *TF* expression was observed in rHD1, respectively. Although *RPLP1* expression level showed a statistically significant negative correlation with the expansion index ((R_s_(14) = −0.4750, *p* = 0.0379), *RPL13A*, *NEIL1*, and *MSH3* did not show any significant correlation (Figure 6B). For rHD7, *TF* gene expression showed a strong positive correlation with both expansion index (R_s_(14) = 0.7143, *p* = 0.0019) and curve-fit range (R_s_(14) = 0.7893, *p* = 0.0004) (Figure 6A,B). *RPLP1* and *RPL13A* showed a statistically significant negative correlation with both expansion index ((R_s_(14) = −0.4786, *p* = 0.0367) and (R_s_(14) = −0.5523, *p* = 0.0175), respectively) and curve-fit range ((R_s_(14) = −0.5893, *p* = 0.0116) and (R_s_(14) = −0.6524, *p* = 0.005) respectively) (Figure 6A,B). No statistically significant correlation was found for *CLU*, *NEIL1*, and *MSH3* (Figure 6A,B).

## 4. Discussion

Peripheral tissues from HD monkeys provide a unique opportunity to study CAG instability in different cell types that share the same genetic background. Previously, we demonstrated age- and CAG repeat size-dependent CAG repeat expansion [47]. This study demonstrates tissue/cell-type and CAG size-dependent CAG repeat expansion (Figure 4). One of the hypotheses of HD pathogenesis is a two-step process. First, the rate of somatic expansion dictates the rate of phenotypic onset. Second, somatic expansion causes cytotoxicity resulting in cell death in the venerable cells. Somatic expansion of the CAG repeat has been well documented in various model systems and humans [13,25,26,27,30,50,51,52,53]. A recent report in human postmortem tissues [53] reveals similar findings as our HD monkeys. Moreover, unlike humans, rHD1 carries four alleles with different CAG repeat sizes (8, 35, 45, and 77Q), which provides a unique opportunity to investigate the correlation between CAG repeat sizes and CAG stability in the same individual.

Behaviorally, rHD1 resembles juvenile-onset HD, while rHD7 is comparable to adult-onset HD [49], which suggests the expression level of *HTT* and the size of the HTT fragment are crucial factors for disease onset and severity [49,55]. Tissue-specific instability observed in caudate and putamen of rHD1 and rHD7 was similar to reports in humans and mice [53,58,61,62]. However, the instability of the liver does not typically exceed that of the caudate and putamen, as we observed in rHD7 [13,23,26,27].

Interestingly, a recent human postmortem study showed high CAG repeat size instability in testis and liver similar to rHD1 [53]. Nonetheless, the caudate, putamen, hippocampus, thalamus, and motor cortex were among the most unstable tissues, as seen in other studies [13,23,24,25]. The cerebellum, prefrontal cortex, and peripheral tissues such as the muscle, heart, adrenal gland, and pancreas of rHD1 were highly unstable, similar to other reports [23,25,27,51,53]. Besides the liver, the testis is the most unstable peripheral tissue.

The use of human polyubiquitin-C promoter in rHD1 resulted in the global expression of mHTT. In contrast, mHTT transgene under the regulation of the human *HTT* promoter in rHD7 was expected to mimic human HTT expression pattern. Although overall high instability was observed in rHD1, heart, muscle, kidney, and cerebellum appeared to be among the most stable, while the testis, liver, thalamus, caudate, and putamen were the most unstable. In rHD7, liver, hippocampus, caudate, and putamen showed high instability (Figure 2B) similar to a human postmortem study [53].

Our previous data show rHD1 lymphocytes at the age of 3 months had 8, 27, 42, and 80Q, and rHD7 lymphocytes had 7 and 65Q at the age of 12 months [47,57]. The most stable tissue in this study, muscle, showed 8, 27, 45, 80, 85, and 90Q in rHD1 while rHD7 had 7 and 66Q (Figure 1 and Tables S1 and S2).

It is interesting to note that HD patients with less than 44 repeats have small tissue-specific differences in instability levels [63,64]. In rHD1and in rHD7, higher instability was observed in alleles with a larger repeat size of over 44Q than in alleles with less than 44Q. A prior longitudinal study on three HD monkeys, including rHD7 that carry the same mutant *HTT* transgene with repeat sizes, ranging between 56Q and 70Q, showed that 62Q might be the threshold of CAG repeat instability leading to large CAG expansion [54]. Current data also demonstrate that around 62Q, the expansion index exponentially increases (Figure 4C). Tissue specificity was demonstrated by both expansion index and curve-fit data (Figure 4A,B). Tissue specificity of CAG repeat expansion begs to investigate gene expression in multiple organs to find genetic modifiers that might be involved in CAG expansion. The Genotype-Tissue Expression (GTEx) allows quick screening of the expression pattern of tissue-specific genes that share a similar trend to CAG repeat expansion [65]. Of the top 100 genes in the four tissues with the highest CAG repeat instability, 35 common genes were identified. Among the 35 common genes, 17 genes were mitochondrial genes and did not show a similar expression trend as our instability data. Of the remaining 18, 4 genes CLU, TF, RPLP1, and RPL13A showed similar expression pattern as instability data (Figure 5). Additionally, the trend of gene expressions that have been associated with HD pathogenesis and CAG repeat expansions, such as huntingtin (HTT), huntingtin-interacting protein 1 (HIP1), 8-oxoguanine DNA glycosylase (OGG1) [61], tumor protein 53 (TP53) [66,67], RE1-silencing transcription factor (REST) [68], nuclear factor kappa B (NF-κB) [69], CREB-binding protein (CBP) [70], forkhead box protein 1 (FOXP1) [71], heat shock factor 1 (HSF1) [72], FANCD2- and FANCI-associated nuclease 1 (FAN1) [62,73], postmeiotic segregation increased 1 homolog 1/2 (PMS1/PMS2) [62], mutL homolog 1/3 (MLH1/MLH3) [62,74], transcription elongation regulator 1 (TCERG1) [62], ribonucleotide reductase regulatory TP53 inducible subunit M2B (RRM2B) [62], coiled-coil domain containing 82 (CCDC82) [62], apurinic/apyrimidinic endodeoxyribonuclease 1 (APEX1) [75], DNA ligase 1 (LIG1) [62], breast cancer 1 (BRCA1) [75], nei like DNA glycosylase 1 (NEIL1) [58], and mutS Homolog 2/3 (MSH2/MSH3) [62,76] in various tissues were analyzed. Only NEIL1 and MSH3 showed similar gene expression patterns as CAG repeat expansion (Figure 6E,F). However, correlation analysis shows a positive correlation between CAG repeat expansion with CLU and TF while a negative correlation between CAG repeats expansion with RPLP1 and RPL13A (Figure 6). Both CLU and TF are involved in oxidative stress response, apoptosis, and DNA damage response (DDR) and have been reported to be involved in Huntington’s disease pathogenesis [77,78,79]. Especially, increased CLU expression has been associated with Alzheimer’s disease, where increased CLU decreased toxicity and the aggregation of amyloid-beta (Aβ) [80]. Additionally, CLU is involved in Aβ aggregation and clearance, neuroinflammation, and regulations of neuronal cell cycle and apoptosis [80]. Moreover, RNA sequence analysis of human Huntington’s disease brain showed CLU as one of the top differentially expressed genes [81]. One study overexpressing CLU in the COS-7 cell line (African green monkey kidney cell) showed the formation of aggresomes, severe interruption of mitochondrial distribution, and triggering of the mitochondria-mediated apoptotic pathway [82], which are the hallmark phenotypes of neurodegenerative disease such as HD and AD. Transferrin gene expression has also been implicated in HD [78]. Iron overloading has been reported in HD models [78] and HD patients [83,84,85]. Moreover, an antibody against transferrin receptor and deferoxamine (iron chelator) has been successfully used in treating HD symptoms [86,87]. However, its impact on CAG repeat size has not been investigated, which warrants further investigation. Both RPLP1 and RPL13A code for ribosomal proteins and are structural components of the ribosome. Although no association with HD has been reported, their involvement in the elongation step of protein synthesis might suggest why they are negatively regulated in tissues with high CAG repeat instability. Interestingly, neither NEIL1 nor MSH3 showed a statistically strong correlation. Although NEIL1 and MSH3 have been reported in GWAS studies [62] and verified by animal studies [15,58,60], most prior studies were focused on the brain and blood samples. Therefore, a comprehensive multi-omics study including genomic, transcriptomic, epigenomic, and proteomic studies on multiple tissues is a critical step to uncover genetic modifiers that affect CAG instability in HD and other TNR expansion diseases. A recent human postmortem study on HTT CAG and ATXN1 CAG expansion showed high tissue-specific CAG expansion between the two genes [53], which suggests a common pathogenic mechanism among TNR expansion diseases and HD monkeys might provide valuable insight to investigate TNR instability and pathogenesis.

It will be pertinent in the future to replicate this study with a larger sample size. Still, our findings suggest that the HD monkey model could contribute significantly to advancing HD research and preclinical studies. rHD7 had distinct tissue-specific instability that largely mirrored human HD. rHD1 contains multiple alleles with differing CAG repeat sizes that might be useful to investigate the effect of CAG repeat sizes on their stability with the same genetic background.

## 5. Conclusions

Our study provides further evidence that CAG repeat expansion is an age-, tissue-, and size-dependent process. This study set the future avenues of investigations that could delineate the biological processes involved in CAG expansion and pathogenesis that can be targeted for future therapeutic development.

## Figures and Tables

**Table 1:** Genotypes of nonhuman primates used in this study.

Genotypes	rWT1	rWT1	rHD1	rHD7
Exons			1	1–10
Promoter			Human polyubiquitin-C	Human *HTT* gene
N-terminal			67 amino acids	508 amino acids
Baseline polyQ length	7, 8Q	7, 8Q	8, 27, 45, 77Q, 87Q	7, 68Q

Both rWT1 and rWT2 had 7Q and 8Q. rHD1 had exon 1, 67 amino acids in the N-terminal, and 8, 27, 45, 77Q, and 87Q due to multiple integrations of the transgene. In contrast, rHD7 had exons 1-10, 508 amino acids in the N-terminal, and 7 and 68Q repeats at the baseline. rHD1 had the ubiquitin promoter, and rHD7 had the human *HTT* promoter. rHD1 showed a much more severe phenotype that resembled juvenile-onset HD. rHD7 had a milder phenotype mirroring adult-onset HD.

## Data Availability

Data is contained within the article and Supplementary Materials.

## Supplementary Materials

The following supporting information can be downloaded at: https://www.mdpi.com/article/10.3390/biomedicines10081863/s1, Figure S1: Electorpherograms of all rWT1 tissue samples; Figure S2: Electorpherograms of all rWT2 tissue samples; Figure S3: Electorpherograms of all rHD1 tissue samples; Figure S4: Electorpherograms of all rHD2 tissue samples; Figure S5: Curve-fit data of rHD1 and rHD7 without endogenous HD TNR allele (8Q); Figure S6: Correlation analysis of expansion index, curve-fit range, and gene expression data from Genotype-Tissue Expression (GTEx). X1 and X2 represent rHD1 expansion index and curve-fit range. X3 and X4 stands for rHD7 expansion index and curve-fit range followed by *ACTB, APOE, CLU, FTL, RPL13A, RPL3, RPLP1, RPS11, RPS12, RPS16, RPS18, PEBP1, C3, IGFBP1, C1R, TF, IFITM3, REEP3, MT-ATP6, MT-ATP8, MT-CO1, MT-CO2, MT-CO3, MT-CYB, MT-ND1, MT-ND2, MT-ND3, MT-ND4, MT-ND4L, MT-ND5, MT-ND6, MT-RNR1, MT-RNR2, MTND2P28;* Table S1: Summary of curve-fit data for rHD1; Table S2: Summary of curve-fit data for rHD7
