# Supplementary material for: CAG Repeat Instability in the Peripheral and Central Nervous System of Transgenic Huntington’s Disease Monkeys
Source: Biomedicines. 2022 Aug 2;10(8):1863. doi: 10.3390/biomedicines10081863 (PMC9405741; doi:10.3390/biomedicines10081863)
Supplement: Supplementary file 1 [file biomedicines-10-01863-s001.zip › Supplemental Tables.pdf]

Supplemental Table S1. Summary of curve-fit data for rHD1.

Tissue	Adrenal	Heart	Kidney	Liver	Lung	Muscle	Pancreas	Testis	Caudate	Cerebellum	Hippocampus	Motor	Prefrontal	Putamen	Thalamus
	8.515	8.032	7.954	8.169	7.967	8.291	7.868	7.959	7.883	7.968	7.961	7.967	7.949	6.86	7.833
	18.9	18.22	18.06	20.67	19.37	11.75	21.18	19.16	13.55	23.47	25.6	25.38	23.78	11.78	22.18
	23.48	25.59	25.36	25.93	25.89	25.62	25.87	26.07	25.33	25.36	25.82	26.65	25.75	18.23	25.53
	37.18	26.83	26.75	26.85	26.9	26.68	26.92	26.73	26.7	26.76	26.59	43.82	26.66	25.47	26.66
	44.01	43.8	43.5	41.46	43.69	40.33	43.63	42.75	33.52	45.93	43.41	44.79	41.05	25.64	44.12
	44.76	44.78	44.76	43.9	44.73	44.29	44.62	42.76	44.44	46.57	44.83	46.82	43.43	33.61	44.7
	47.1	44.82	47.17	44.69	46.14	44.63	64.36	44.63	47.82	50.18	47.1	81.27	44.83	44.09	48.65
	63.89	47.14	49.16	46.49	76.14	47.1	66.32	45.4	52.12	50.24	68.47	71.22	47.01	44.73	71.72
	78.72	60.19	66.16	71.87	77.65	68.13	79.09	75.92	61.43	74.46	80.1	80.07	72.67	47.19	80.2
	80.41	75.96	79.51	79.84	89.42	76.95	85.17	76.66	68.04	78.21	80.28	87.93	80.04	52.51	88.04
	85.67	80.15	87.92	89.89	90.62	80.26	90.05	86.56	80.28	87.45	87.34	90.19	87.75	79.95	90.14
	90.15	85.9	90.33	90.7	84.51	85.67	96.26	90.15	85.14	88.26	90.05	76.98	90.17	87.71	56.17
	93.45	90.16		70.9	114.8	90.12	105.5	104.4	88.89	91.14	58.37	125	29.28	88.87	96.76
	110.7	84.44		92.45				126.3	90.52	92.45	123.3		112.2	90.51	141
	119.8	94.19		105.9				138.7	92.37	97.4			127.2	97.26	
	136.6			150.2				146.6	96.51					129.3	
								159.7	116.9					136.5	
									143.4						
Average Error	1.9075	1.4803	1.0576	1.3557	1.7462	1.4023	0.9855	1.923	1.016	0.373	1.383	2.0893	1.3397	0.186	95.07
Average R^2	0.9938	0.9948	0.9975	0.9957	0.9934	0.9966	0.9975	0.9885	0.9914	0.9991	0.9882	0.9922	0.9952	0.9997	0.9345

Supplemental Table S2. Summary of curve-fit data for rHD7.

Tissue	Adrenal	Caudate	Cerebellum	Heart	Hippocampus	Kidney	Liver	Lung	Motor	Muscle	Pancreas	Prefrontal	Putamen	Testis	Thalamus
	7.536	7.307	7.441	7.413	7.402	7.539	7.354	7.497	7.364	7.355	7.56	7.398	7.402	7.385	7.352
	23.54	34.84	28.15	21.05	14.63	25.64	26.78	21.4	25.06	23.72	30.05	27.09	33.58	16.11	10.79
	65.53	68.67	68.12	66.76	24.22	67.85	67.56	67.91	61.12	70.85	68.03	67.38	69.02	29.7	31.71
	67.78	72.08	69.18	67.72	26.45	69.42	71.29	69.69	68.55		69.39	70.78	71.87	40.84	68.99
	69.45	82.93	69.65	71.74	35.83	71.73	75.19		71.11		69.44	76.04	73.04	47.98	71.78
		93.16			69.07		80.05		75.14				76.19	64.51	75.08
		97.62			73.72		94.74						94.74	67.44	
		116.5			79.13		108						102.2	69.1	
		141.5			87.74		137.3						112.8	72.35	
					95.83								137	76.29	
					140.9									79.49	
Average Error	0.779	2.048	0.318	0.918	3.5525	0.528	2.5325	0.547	0.402	0.306	0.419	0.923	2.451	0.365	0.489
Average R2	0.9981	0.9912	0.9998	0.9971	0.9794	0.9994	0.9893	0.9991	0.9997	0.9994	0.9995	0.9954	0.9907	0.9996	0.9995
