# Supplementary figures and images for: CAG Repeat Instability in the Peripheral and Central Nervous System of Transgenic Huntington’s Disease Monkeys
Source: Biomedicines. 2022 Aug 2;10(8):1863. doi: 10.3390/biomedicines10081863 (PMC9405741; doi:10.3390/biomedicines10081863)

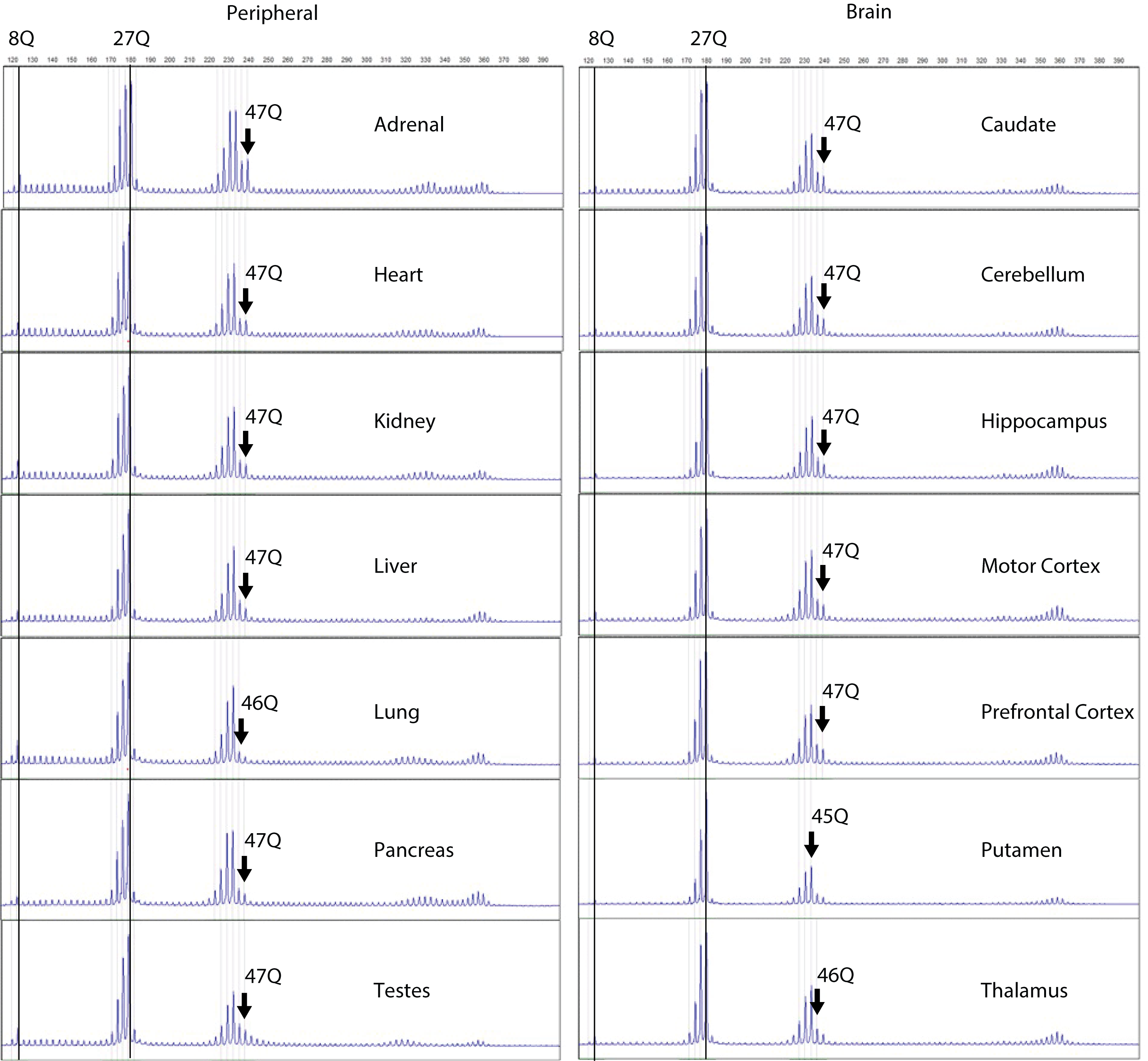

Supplement: Supplementary file 1 [file biomedicines-10-01863-s001.zip › Figure S3.tif]

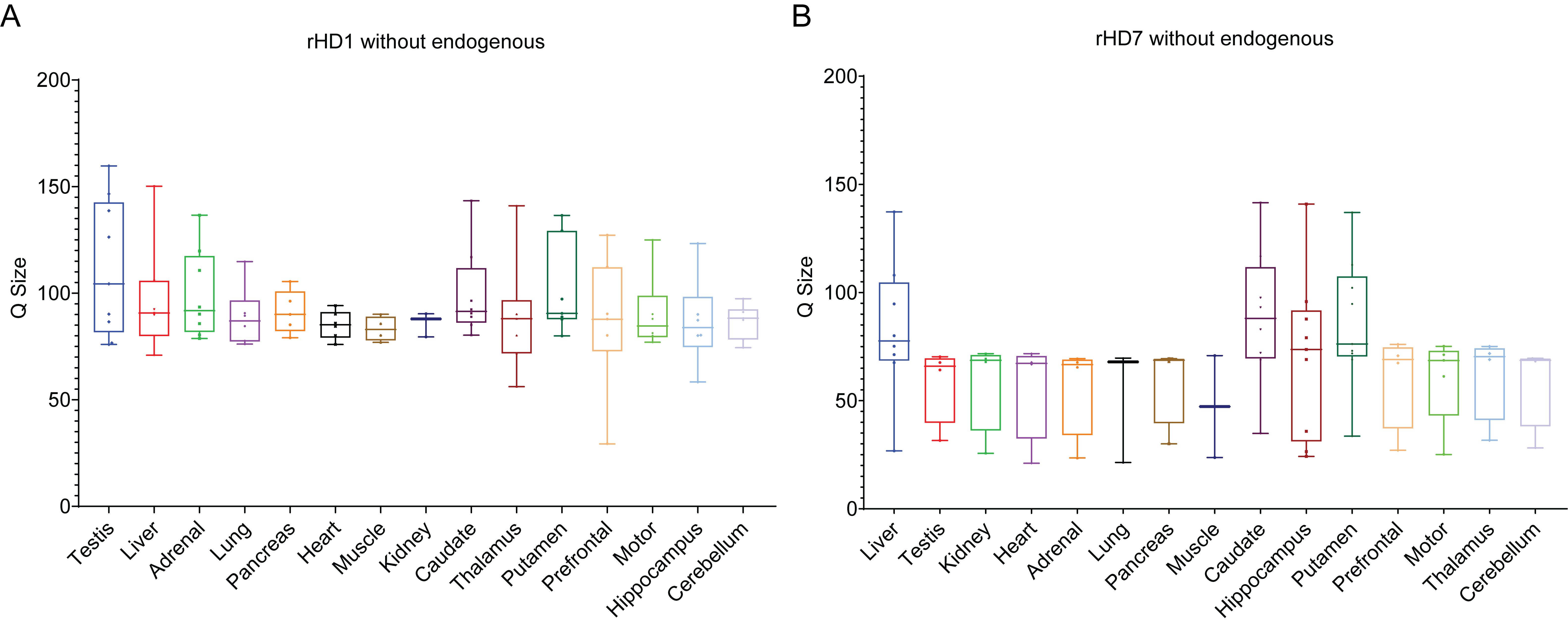

Supplement: Supplementary file 1 [file biomedicines-10-01863-s001.zip › Figure S5.tif]

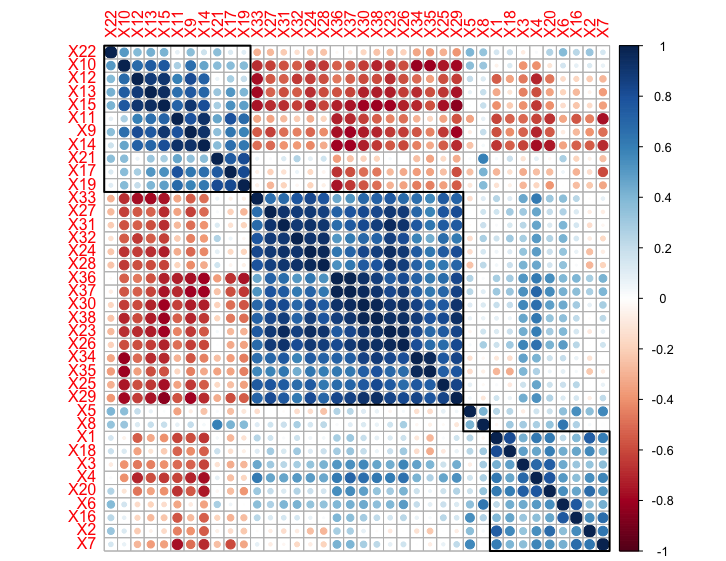

Supplement: Supplementary file 1 [file biomedicines-10-01863-s001.zip › Figure S6.png]
